# *In ovo* model in cancer research and tumor immunology

**DOI:** 10.3389/fimmu.2022.1006064

**Published:** 2022-09-29

**Authors:** Lea Miebach, Julia Berner, Sander Bekeschus

**Affiliations:** ^1^ ZIK plasmatis, Leibniz Institute for Plasma Science and Technology (INP), Greifswald, Germany; ^2^ Department of General, Thoracic, Vascular, and Visceral Surgery, Greifswald University Medical Center, Greifswald, Germany; ^3^ Department of Oral and Maxillofacial Surgery, Plastic Surgery, Greifswald University Medical Center, Greifswald, Germany

**Keywords:** CAM, cancer immunity, patient-derived xenografts, oncology, macrophages

## Abstract

Considering cancer not only as malignant cells on their own but as a complex disease in which tumor cells interact and communicate with their microenvironment has motivated the establishment of clinically relevant 3D models in past years. Technological advances gave rise to novel bioengineered models, improved organoid systems, and microfabrication approaches, increasing scientific importance in preclinical research. Notwithstanding, mammalian *in vivo* models remain closest to mimic the patient’s situation but are limited by cost, time, and ethical constraints. Herein, the *in ovo* model bridges the gap as an advanced model for basic and translational cancer research without the need for ethical approval. With the avian embryo being a naturally immunodeficient host, tumor cells and primary tissues can be engrafted on the vascularized chorioallantoic membrane (CAM) with high efficiencies regardless of species-specific restrictions. The extraembryonic membranes are connected to the embryo through a continuous circulatory system, readily accessible for manipulation or longitudinal monitoring of tumor growth, metastasis, angiogenesis, and matrix remodeling. However, its applicability in immunoncological research is largely underexplored. Dual engrafting of malignant and immune cells could provide a platform to study tumor-immune cell interactions in a complex, heterogenic and dynamic microenvironment with high reproducibility. With some caveats to keep in mind, versatile methods for *in* and *ex ovo* monitoring of cellular and molecular dynamics already established *in ovo* are applicable alike. In this view, the present review aims to emphasize and discuss opportunities and limitations of the chicken embryo model for pre-clinical research in cancer and cancer immunology.

## Introduction

As early as 1911, Rous and Murphy were the first to transplant primary tumor tissue on the highly vascularized chicken embryo CAM, demonstrating the rapid growth of Rous sarcomas early after engrafting. Starting from those findings, the *in ovo* model gained emerging interest as an alternative for costly, time-consuming mammalian *in vivo* models in pre-clinical oncological research (1). For centuries a drawback in rodent models, tumor cells and tissues can be engrafted at high efficiencies without species-specific restrictions due to the embryo’s natural immunodeficiency (2, 3). By that oncogenesis can be studied in a humanized system with widespread adoption. Developing tumors perform neo-vascularization and matrix deposition, mimicking the complex tumor microenvironment largely limited in conventional *in vitro* models (4). The extraembryonic membranes are connected to the embryo through a continuous circulatory system, readily accessible for manipulation and versatile visualization techniques to study tumor growth, metastasis and angiogenesis *in* and *ex ovo*, deep mechanistic insights at cellular and molecular levels can be achieved after tumor excision. Ethical approval is omitted if experiments are terminated at embryonic day 14 in most countries, facilitating screenings of pharmacological or physics-based therapies with high reproducibility at large scales supporting the 3Rs principle (5).

In spite of a valuable history in pre-clinical oncology, the applicability of the tumor chorioallantoic membrane (TUM-CAM) model in immunoncological research is largely underexplored. Only few studies have taken advantage of a naturally immunodeficient host to study tumor-immune interactions without species-specific restrictions *in ovo.* Rodent models have yielded fundamental insights into key aspects of the human immune system, including its dual role in elimination and guarding of malignant cells. As such, the discovery that culture supernatants of activated T cells can boost the reactivity of previously generated cytotoxic T lymphocytes gave rise to the first, IL-2 based, immunotherapy already 30 years ago (6–8) and new waves of excitement are dedicated to recent advances in antibody and cell based therapy approaches. On the other hand, it is undisputable that cancer cells are excellent maskers, hijacking and coopting immune and stromal cells residing in their microenvironment to aid in tumor progression and metastasis. Tumor-immune cell interactions evolve in a highly dynamic, heterogenic environment, and mammalian models remain state-of-the-art reflecting the patient’s situation. However, establishment of clinically relevant, cost and time efficient 3D models for basic and translational immunooncological research at larger scales is urgently needed. The TUM-CAM model is ideal to bridge this gap.

The present review aims to summarize, highlight and emphasize opportunities and limitations of the chicken embryo model for its use in pre-clinical (immuno-)oncological research. Moreover, its potential for tailored treatments based on patient-derived xenografts (PDX) in the context of personalized medicine is discussed.

### The *in ovo* model in cancer research

The *in ovo* model has served as an alternative for mammalian tumor models to investigate characteristics of tumor growth, metastasis, and efficacy of cancer therapies in preclinical oncological research for more than a century. Grafting of tumor cell suspensions but also murine and human primary tissues has been successfully applied to address a broad range of scientific questions with diverse methodological read-outs (**Table 1**).

**Table 1:** Advantages and limitations of methods that have been established *in ovo*.

Readout	Purpose (examples)	Requirements	Advantages	Limitations		Reference (DOI)
**macroscopic**						
**weight**	- tumor mass - tumor volume	- precision scale	- easy feasibility - high throughput - low costs	- unprecise and volatile (surrounding stroma) - insensitive		(9–12)
**TIVITA**	- angiogenesis - health status - tissue oximetry	-TIVITA camera and computer	- easy feasibility - noninvasive - monitoring at multiple timepoints - assessment and quantification of tissue-specific parameters (e.g. StO2, THI) *in vivo*	- time-consuming evaluation - manual evaluation imprecise		(13)
**stereo microscope**	- tumor growth - tracking of labeled cells - angiogenesis	- stereomicroscope	- easy feasibility - noninvasive - monitoring at multiple timepoints *in vivo*	- costly equipment		(14–16)
**microplate reader**	- tumor growth - viability/metabolic activity - cell death kinetics - gene expression *via* reporter genes	- luciferase-transduced/fluorescent cells - Resazurin/WST-1/MTT - microplate reader	- precise determination of (viable) tumor mass *ex vivo* - high throughput	- requires inoculation of luciferase-transduced/ fluorescent cells		–
**luminescence**	- tumor growth - gene expression *via* reporter genes	- luciferase-transduced cells - chemiluminescent substrate - imager	- monitoring of tumor growth at multiple timepoints - noninvasive - precise determination of viable tumor mass *in vivo*	- requires inoculation of luciferase-transduced cells - costly equipment		(12, 13, 17–19)
**MRI**	- tumor growth	- MRI and equipment	- noninvasive - monitoring of tumor growth at multiple timepoints - assessment of biodistribution of labelled compounds	- time-consuming - costly equipment		(11, 20–22)
**ultrasonography**	- repetitive visualization of tumor growth and vascularization - evaluation of anti-angiogenic therapy response	- ultrasonographic scanner/ultrasound devicetab	- cost efficient - longitudinal monitoring of tumoral development - easily applicable	- interpretation depends on operator and manually adjusted machine settings		(23–25)
**cellular**						
**FACS**	- toxicity - differentiation - ICD - ROS levels	- cytometer - tissue dissociator - lysis buffer - strainer - antibodies	- enables broad range of downstream assays - analysis of intra- and extracellular marker expression - high throughput - examination of cancer-immune cell interactions	- samples are sticky - sample heterogeneity - requires equipment for tumor digestion		(13)
**sections**	- apoptosis - EMT - differentiation - immune landscape - invasion of cancer cells	- kryotome/microtome - antibodies - microscope	- enables visualization of immune cell infiltration	- time consuming sample preparation - quantitative image analysis time consuming and high knowledge level		(26–29)
**subcellular**						
**ELISA**	- growth factors - immunogenicity - angiogenesis	- ELISA - bead-based multiplex assay	- species specificity - high-throughput - examination of cancer-immune cell interactions	- multiplex assay costly - tissue sampling after tissue dissociation?		–
**PCR/** **transcriptomics**	- underlying molecular mechanisms - metastatic potential - spread of oncolytic viruses	- tissue dissociator - RNA isolation kit	- underlying mechanisms	- tissue sampling - low DNA/RNA yield		(30–32)
**proteomics/** **lipidomics**	- protein expression analysis - metabolomics profiling	- tissue dissociator - mass spectrometer - protein profiler array	- sensitivity	- low protein yield - high cost and technical demand (MS) - time-consuming (MS)		(33, 34)
**WB**	- protein expression analysis	- tissue dissociator - western blot equipment	- underlying mechanisms - sensitivity and specificity	- low protein yield - prone to false/subjective results - high cost and technical demand		(33, 35)

### General experimental procedure and ethical guidelines

Several techniques have been established to engraft tumor cells and tissues on the vascularized CAM, with main differences between *in ovo* and shell-less *ex ovo* approaches. The latter offers better accessibility of the CAM but increases drop-out rates due to frequent rupture of the yolk membrane and contamination, requiring high levels of experience (36, 37). With respect to its prevalent use in cancer research, the present review provides a step-by-step protocol of the general experimental procedure *in ovo.*

**Figure 1:**
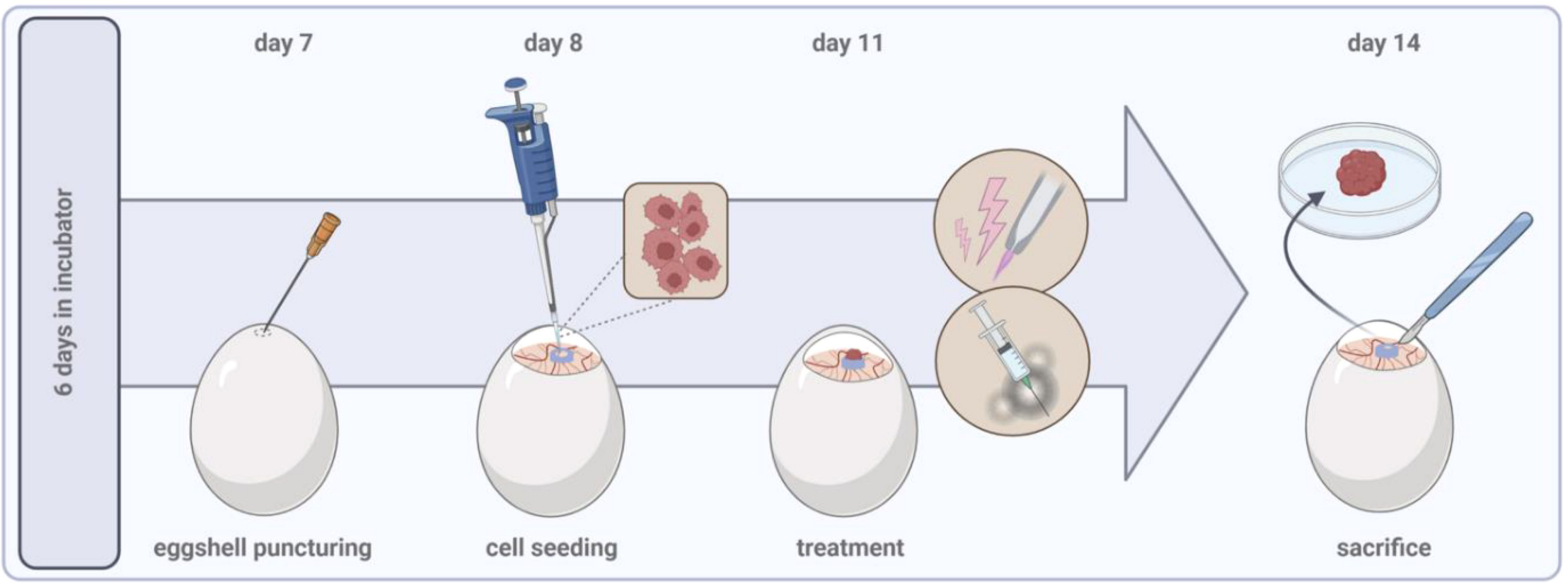
General experimental procedure of the tumor chorioallantoic membrane model.

Specific pathogen-free (SPF) fertilized chicken eggs are placed in a specialized breeding incubator equipped with a turning unit in horizontal position. Commercial hatcheries turn eggs once every hour but should be at least 3 times a day to prevent embryo adhesion to the shell. Breeding conditions should be at 37°C and 65% humidity throughout the experimental period. After six days of incubation, the pointed pole of the eggs can be punctured using a 20 G cannula to create an air hole for subsequent tumor inoculation. The hole is covered with an air permeable plaster, and eggs are placed back in the static unit of the incubator, now in vertical position. Prior puncturing, fertilization, and viability can be easily verified by candling the egg using a bright flashlight. Unfertilized, dead, or contaminated eggs should be removed at any time throughout the experimental period to avoid cross-contaminations. Sterile conditions are not necessarily needed for any intervention but might help to preserve high viability during long-term incubation due to reduced infection risks. On day 7, the egg shell is windowed at the punctured side with a diameter of approximately 2 cm. Caution should be drawn to the highly vascularized CAM. A larger hole can facilitate tumor inoculation and, e.g., *in vivo* monitoring of tumor growth, but raises the risk of damaging the CAM and reducing viability rates due to severe bleeding and contaminations. Next, a filter paper of the size of approximately 1 cm x 1 cm is soaked in diethylether and placed shortly (≤ 1s) central on the visible CAM as local vessel arrosion has been shown to facilitate tumor formation (38, 39). Immediately after, tumor cells can be inoculated in a silicon ring (inner diameter ≥ 5 mm) placed on the CAM. Engrafting of 1-2 mio. cells per egg is commonly reported in the literature, but a titration to optimize numbers specifically for different cell lines is recommended (**Table 2**). Solid tumor formation of cell suspensions is improved if cells are resuspended in a hydrogel mixture. A pre-manufactured gel is easy to handle while minimizing basal toxicity, but self-made, e.g., collagen mixtures are feasible alike (9). Again, the hole is covered with a transparent, air-permeable foil, and eggs are incubated for a minimum of three days to allow solid tumor formation prior to treatment on day 11. The tumor-chorioallantoic membrane (TUM-CAM) model has been investigated as a benchmark model for an array of oncological therapies, including various chemotherapeutics (44, 45), targeted (23) and checkpoint therapies (46), oncolytic viruses (30), radiotherapy (47), photodynamic therapy (48), and medical gas plasmas (49). Administration of single and several therapy cycles paralleled by continuous monitoring of tumor growth (*view chapter 2.2*) is feasible. By that, the TUM-CAM model is a versatile but likewise relatively simple, low-cost model that allows screening of pharmacological or physics-based therapies in a short time. Embryos are sacrificed on day 14, and excised tumors and additional tissue samples can be analyzed in various versatile downstream assays (**Figure 1**).

**Table 2:** List of cell lines that have been engrafted *in ovo*.

Cell line^laboratory^	Entity	Species	Seeding number	Tumor weight (mg)	Days p.i.	Reference (DOI)
**A431^1^**	squamous cell	human	1 x 10^6^	46.1	7	unpublished
**A549^2^**	lung	human	3 x 10^6^	33.0	10	(32)
**A375^3^**	melanoma	human	2 x 10^6^	34.0	7	(40)
**B16F10-luc^1^**	melanoma	murine	1 x 10^6^	36.0	6	unpublished
**CT26-luc^1^**	colon	murine	1 x 10^6^	30.6	6	(13)
**CT26-luc^1^**	colon	murine	2 x 10^6^	32.6	6	(12)
**HT29^1^**	colon	human	1 x 10^6^	35.2	6	unpublished
**HT1080^4^**	fibrosarcoma	human	8 x 10^4^	64.0	7	(31)
**KPC960GFP-luc^1^**	pancreatic	murine	1 x 10^6^	25.6	7	unpublished
**MC38^1^**	colon	human	1 x 10^6^	6.60	7	unpublished
**MDA-MB-231^5^**	breast	human	2 x 10^6^	5.10	9	(21)
**MDA-MB-231^6^**	breast	human	1 x 10^6^	74.0	9	(34)
**MiaPaca^7^**	pancreatic	human	2 x 10^6^	93.3	6	(41)
**MiaPaca^3^**	pancreatic	human	2 x 10^6^	14.0	5	(42)
**MiaPaca^3^**	pancreatic	human	2 x 10^6^	52.0	7	(42)
**MiaPaca^1^**	pancreatic	human	2 x 10^6^	33.0	6	(10)
**Panc01^1^**	pancreatic	human	1 x 10^6^	39.3	6	unpublished
**Panc01^1^**	pancreatic	human	2 x 10^6^	27.5	6	(10)
**PaTuS^7^**	pancreatic	human	2 x 10^6^	105	6	(41)
**PaTuS^1^**	pancreatic	human	2 x 10^6^	24.0	6	(10)
**PaTuT^1^**	pancreatic	human	2 x 10^6^	30.5	6	(10)
**PDA6606^7^**	pancreatic	murine	2 x 10^6^	37.5	3	(43)
**PDA6606^7^**	pancreatic	murine	2 x 10^6^	35.0	6	(11)
**RB-355^8^**	retinoblastoma	human	1 x 10^6^	50.0	7	(44)
**RT-112^1^**	urothelial	human	1 x 10^6^	9.72	7	unpublished
**RLT-PSC^3^**	stellate cells	human	2 x 10^6^	8.00	5	(42)
**RLT-PSC^3^**	stellate cells	human	2 x 10^6^	13.0	7	(42)
**SCaBER^1^**	urothelial	human	1 x 10^6^	23.6	7	unpublished
**SCC7-luc^1^**	squamous cell	murine	2 x 10^6^	36.0	6	(12)
**SKOV3^1^**	ovarian	human	1 x 10^6^	39.9	6	unpublished
**T24^1^**	urothelial	human	1 x 10^6^	20.0	7	unpublished
**UKF-NB-4^9^**	neuroblastoma	human	1 x 10^6^	70.0	7	(45)
**WERI-Rb1^8^**	retinoblastoma	human	1 x 10^6^	52.0	7	(44)
**Y-79^8^**	retinoblastoma	human	1 x 10^6^	62.0	7	(44)

^1^ ZIK plasmatis, Leibniz Institute for Plasma Science and Technology (INP), Felix-Hausdorff-Str. 2, 17489 Greifswald, Germany; ^2^ Cancer Target and Experimental Therapeutics, Institute for Advanced Biosciences, INSERM U1209, CNRS UMR5309, Grenoble Alpes University, Grenoble, France; ^3^ Research Group PLASMANT, Department of Chemistry, University of Antwerp, BE2610 Wilrijk-Antwerp, Belgium; ^4^ Department of Laboratory Medicine and Pathobiology, Faculty of Medicine, University of Toronto, Toronto, ON M5S 1A1, Canada; ^5^ Department of Internal Medicine II, University Hospital Ulm, Ulm, Germany; ^6^ Department of Physiology and Biophysics, Weill Cornell Medicine—Qatar, Doha 24144, Qatar; ^7^ Department of General, Visceral, Thoracic, and Vascular Surgery, Greifswald University Medical Center, 17475 Greifswald, Germany; ^8^ Institute of Anatomy II, Department of Neuroanatomy, University of Duisburg-Essen, Germany; ^9^ Research Group for Molecular Biology and Nanomedicine, Department of Chemistry and Biochemistry, Mendel University in Brno, Brno, Czechia.

As the chicken embryo is not considered as living animal until day 17 of ontogenesis, the TUM-CAM model does not require administrative procedures for obtaining ethical approval obligatory for common animal models. Experiments are terminated before development of regions in the central nervous system that are associated with experience of pain. The National Institute of Health (50), as well as the Institutional Animal Care and Use Committee (IACUC) (51), established that chick embryos lack pain perception before reaching day 14 of gestation and can therefore be used without any ethical restriction.

### Monitoring of tumor growth, invasion, and metastasis

**Figure 2:**
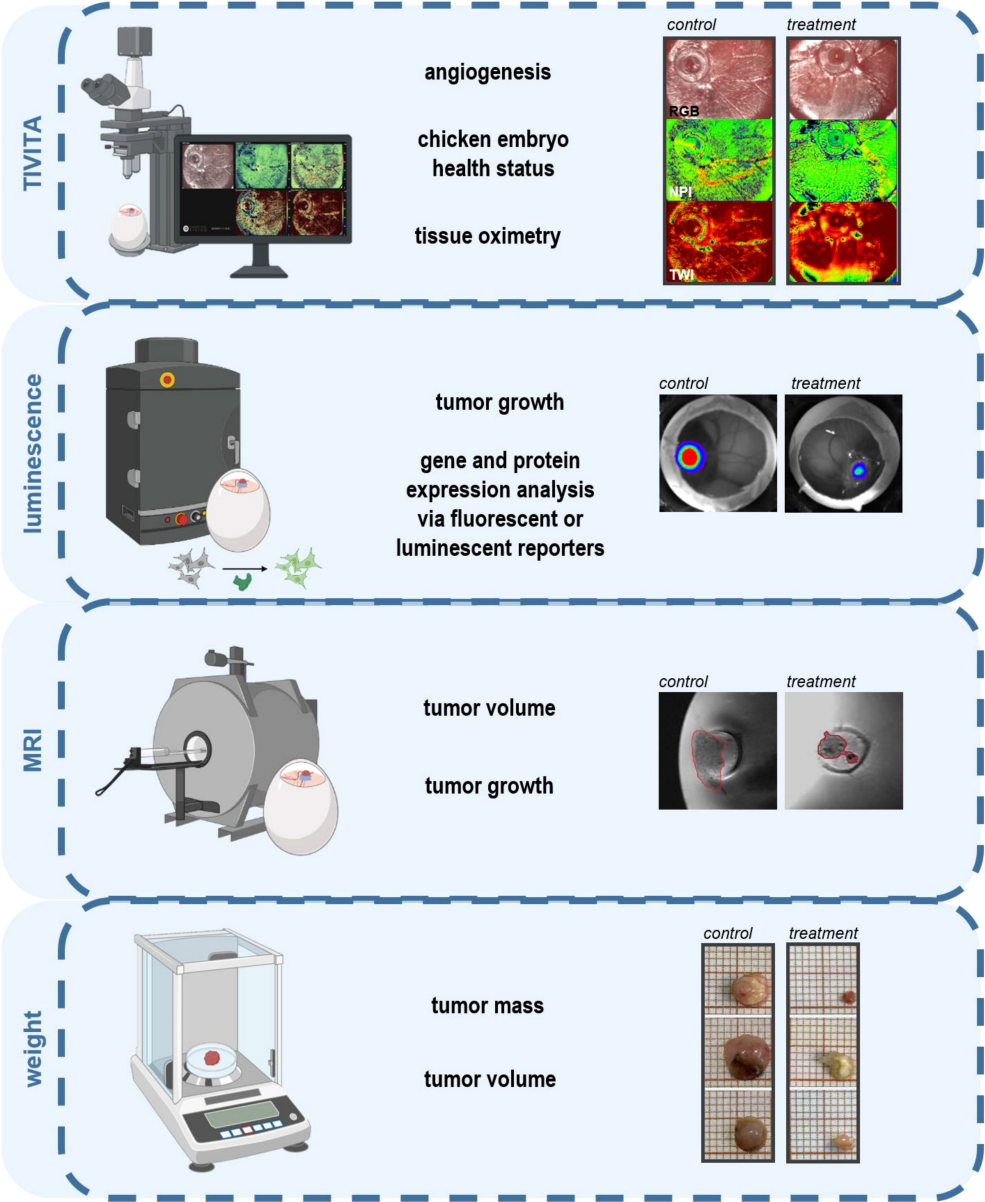
Intravital monitoring of angiogenesis, tumor growth, and assessment of tumor weight (11, 13).

Tumor growth as the primary objective in screening studies or mechanistic knock-out models has been assessed by endpoint analysis by means of size, volume, or tumor weight measurements after excision at the simplest. As a major limitation, repeated tumor monitoring is unfeasible in this case. More versatile methods applicable also for longitudinal monitoring include two- or three-dimensional imaging or the evaluation of surrogate parameters based on bioluminescence. The latter requires grafting of genetically engineered cell lines equipped with luciferase reporter genes (17). Upon administration of the respective substrate luciferin, luciferase-expressing tumor cells emit light, allowing to evaluate the tumor growth as a linear function of light emission detected with appropriate optical detection systems. Due to excellent light penetration and biodistribution of the substrate, bioluminescent imaging could also serve to investigate distant metastasis in chick embryo organs but might be limited to shell-less *ex ovo* approaches. Along similar lines, fluorescent-tagged tumor cells have been engrafted (14, 52). Three-dimensional visualization of tumor development has been achieved using advanced imaging techniques such as micro-computed tomography (CT) (53) and magnetic resonance imaging (MRI) (20). However, time expenditure, high costs, and technical demand are obvious limitations. In addition, the high density of the egg shell causes radio-opacity with obligate need for contrast agent administration (54). High levels of experience are needed for intravascular injections in the small CAM vessels due to a high risk of excessive bleeding, causing increased drop-out rates. Several research groups have focused on noninvasive, three-dimensional imaging such as repeated ultrasonography (24). If accessible, this imaging technology has low operating costs and is time-efficient without need for application of additional imaging agents (**Figure 2**).

As the extraembryonic membranes are connected with the avian embryo through a continuous circulatory system readily accessible for manipulation, the *in ovo* model represents a comprehensive experimental system that recapitulates all steps in the metastatic cascade. While available 3D *in vitro* models mimic only poorly the structure of blood vessels, particularly small vessels such as capillaries and post-capillary venules, where cancer invasion is believed to take place, rodent models are often limited by the scarcity of metastasizing tumors. The highly vascularized nature of the CAM provides by contrast a platform to study both experimental and spontaneous metastasis. Cancer cell invasion, extravasation, and metastatic colonization have been investigated *in ovo* by morphological assessment (55), selective outgrowth of individual metastasized cells, and detection of microscopic tumor colonies (56). Moreover, biomarkers including human urokinase plasminogen activator (uPA) activity (57) and others related to epithelial-mesenchymal transition (EMT) have been evaluated using various methods, including enzyme-linked immunosorbent assay (ELISA), tissue staining, fluorescence-activated cell sorting (FACS) and polymerase chain reaction (PCR) (26). Cancer cell metastasis based on tumor cell grafting on the CAM has provided valuable information regarding the complex series of events involved in the metastatic cascade. After penetration of the chorionic epithelium, invasion of the mesenchyme below, and blood vessels (58), tumor cells have been shown to survive in the circulation (59), arrest in the vasculature, and proliferate in distinct organs after extravasation (60, 61). As a major drawback in standard mouse models of experimental metastasis, the majority of injected tumor cells perishes in the microcirculation before extravasation can be observed. In the chicken CAM metastasis model, 80% of tumor cells injected in the allantoic vein have been shown to survive and extravasate after 1-3 days following migration to the vicinity of preexisting vessels (62). Longitudinal monitoring has been achieved by establishing an intravital microscopy platform for high-resolution time-lapse imagery of human tumor growth, cell migration, and extravasation *in ovo*. After luminal labeling of avian endothelial cells *via* intravenous injection of fluorescent *Lens culinaris* agglutinin and visualization of blood flow using fluorescent dextran, fluorescent human epidermoid carcinoma Hep3-GFP cells were injected into the vitelline vein to visualize tumor cell extravasation and the role of invadopodia in metastatic processes over a 24 h period (60). Spontaneous metastasis after tumor cell grafting of the CAM has been investigated, e.g., by injection of labeled cells that can be traced in organs, blood, and amnion *in* and *ex ovo* (63). In a study investigating two isogenic fibrosarcoma cell lines with differing intravasation capacities, HT-1080-hi/diss, and HT-1080–lo/diss, topically applied highly disseminating cells appeared localized under the CAM ectoderm already 24 h after inoculation (64). Circulating cancer cells have been detected using fiber optic array scanning technology (FAST). Therefore, peripheral blood was distributed on glass slides and immunostained with anti-human CD44 mAb 29-7. Invasion of tumor cells in the circulatory system was first detected 3 days after tumor cell grafting with the highest frequency observed on day 4 (65), which coincides with the onset of intravasation determined in the distal CAM by qPCR (64). Given proof of metastatic colonization in distinct organs is partially limited by the low amount of tumor cells that needs to be detected. Most tumor cells are not able to produce macroscopic visible colonies within the short experimental time. However, as the human genome is uniquely enriched in Alu sequences (frequency 5%), metastasized human tumor cells can be detected and quantified by sensitive Alu PCR assays (66, 67) which enables detection quantification of a minimum of 50 cells (61).

### Tumor angiogenesis and vascular remodeling

Expanding tumors hijack physiological angiogenesis to deliver adequate oxygen and nutrient supply, enable waste disposal, and facilitate dissemination of cancer cells to distant sides. During the avascular phase early in tumor progression, the tumor size is largely limited to 1-2 mm. Nutrient deprivation can trigger an ‘‘angiogenic switch’’, resulting in vascular branching and proliferation of endothelial cells, enabling the tumor to grow beyond a restricted size by ensuring sustained energy supply (68). Deregulation of angiogenesis as a hallmark of cancer (69) plays a major role in disease progression, and inhibition of tumor angiogenesis was introduced as a therapeutic strategy more than 50 years ago (70). In 2003, a clinical trial demonstrated prolonged survival of colorectal cancer patients in combination regimes with humanized neutralizing antibodies (bevacizumab, approved by FDA 2005) targeting vascular endothelial growth factor (VEGF), providing proof-of-concept of the successful use of anti-angiogenic therapies in oncology. Likewise, several tyrosine kinase inhibitors designed to target pro-angiogenetic signaling (e.g., sunitinib, approved by FDA 2006) are applied in the treatment of gastrointestinal neoplasms, renal cell carcinoma, and glioblastoma at the front line setting. As a major drawback, hypoxia in the context of aberrant perfusion has been linked to increased resistance to conventional chemotherapeutics, radiotherapy, and immunotherapy, raising the need to unravel further signaling pathways and molecules involved in vascular remodeling and characteristics of vascular structure in the tumor microenvironment.

Conventional *in vitro* assays, including the aortic ring or endothelial tube forming model, have contributed significantly to underlying mechanisms of (patho-) physiological angiogenesis but are limited in reflecting heterogeneous and malformed vessels building the tumor microvasculature, as well as the complexity of the TME. The highly vascularized CAM as the avian gas exchange organ is considered ideal for angiogenic studies. Pharmacological screenings of pro- and anti-angiogenic compounds have long been performed *in ovo*, with emerging interest in its use in angiogenic research in pre-clinical oncology. After tumor inoculation, tumor xenografts become visible within 2-3 days and are readily supplied with blood vessels of CAM origin that penetrate deep into the tissue. Several qualitative and quantitative approaches have been described to evaluate angiogenic responses *in ovo*. Recent advances in imaging technologies, as well as improvements in contrast and imaging agents, allow for visualization of vascular perfusion and selective labeling of vascular structures at microscopic levels. Histological and immunohistochemical analyses are considered the reference standard, including hematoxylin-eosin (HE) stainings, staining of vascular and endothelial markers such as CD31 (47) and von Willebrand factor (vWF) (71), or highlighting the vasculature *via* injection of *Sambuco negro* agglutinin (SNA) which binds specifically to chicken endothelium (72). *In ovo* polymer injection followed by micro-CT has moreover been described for three-dimensional vascular remodeling (71). Besides endpoint analyses, versatile methods have been employed for longitudinal monitoring of vascular dynamics. Under controlled light conditions, digital images can be taken and analyzed using image analysis platforms such as Image J (73). Viral nanoparticles have been used for intravital monitoring of vascular structures and fluid dynamics to a depth of 500 µm over 72 h and were retained even after subsequent pathological tissue analysis (74). Hyperspectral imaging has been applied (13) to quantify superficial and deeper tissue oxygenation related to hemoglobin content and water content in tissues based on mathematical modeling of spectral band intensities (**Figure 2**). Without the need for intravascular application of imaging agents, pre-described ultrasonography can be extended by Doppler mode measurements of vascular structure (24), however largely limited to monitoring of large vessels due to tissue clutter. A more comprehensive characterization of slow-flow vessels in microvasculature has been achieved by high-frequency ultrasensitive ultrasound microvessel (UMI) imaging and validated after administration of two FDA-approved anti-angiogenic agents in a model of renal cell carcinoma (23). As a major limitation, distinguishing tumor-related neoangiogenesis from innate embryonic neovascularization or increased vascular density due to rearrangement of existing vessels can affect data interpretation (62). Timing of angiogenic studies is crucial to avoid respective confounding factors. As the endothelial mitotic index and the general complexity of the CAM decreases around embryonic day (ED) 11, angiogenic studies are widely performed between ED10/11 until ED14/15 (1).

### Further perspectives: Investigating the hallmarks of cancer at cellular and molecular levels

**Figure 3:**
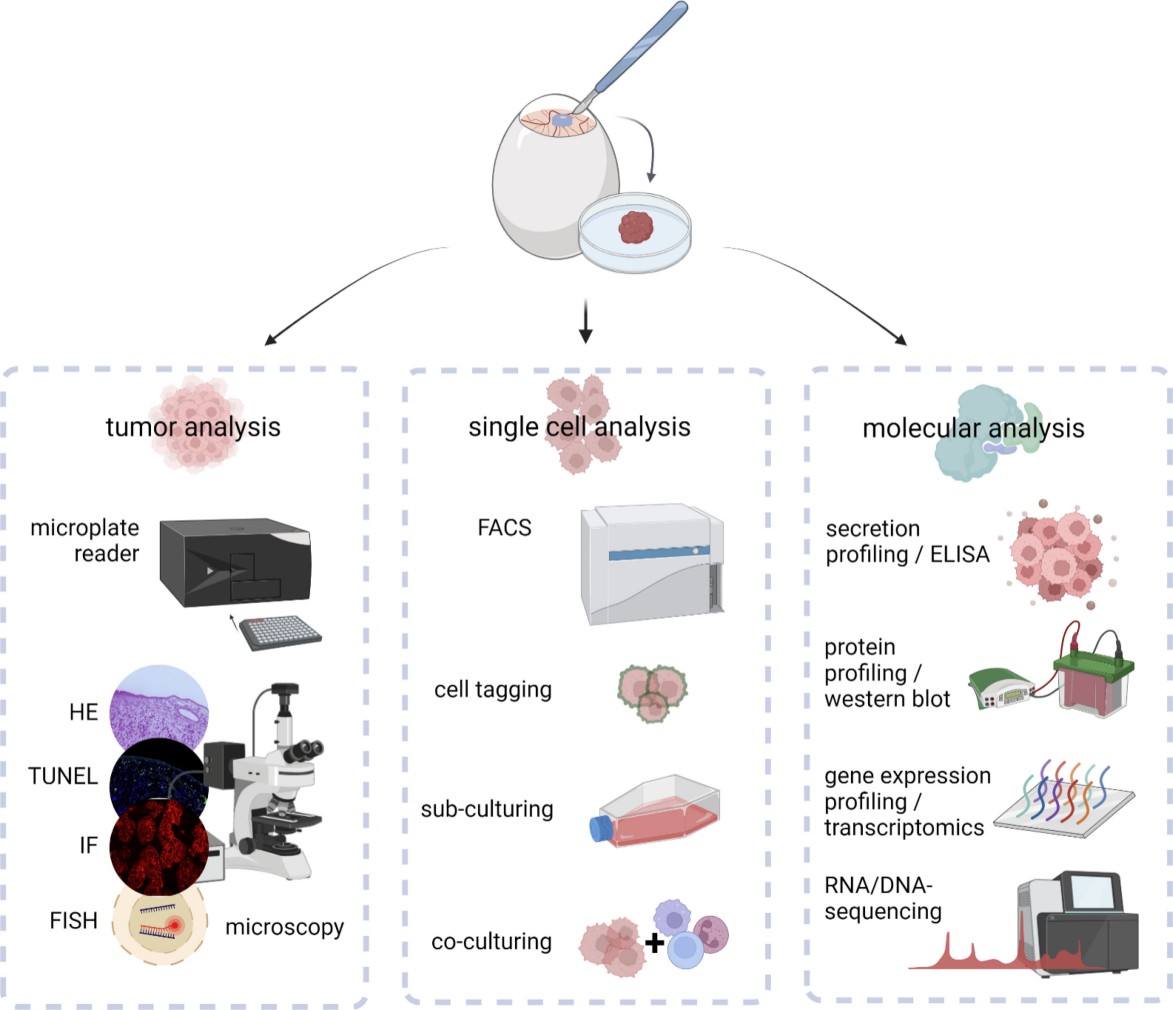
Downstream analysis of excised *in ovo-*grown tumors at macroscopic, cellular, and molecular levels.

**Figure 4:**
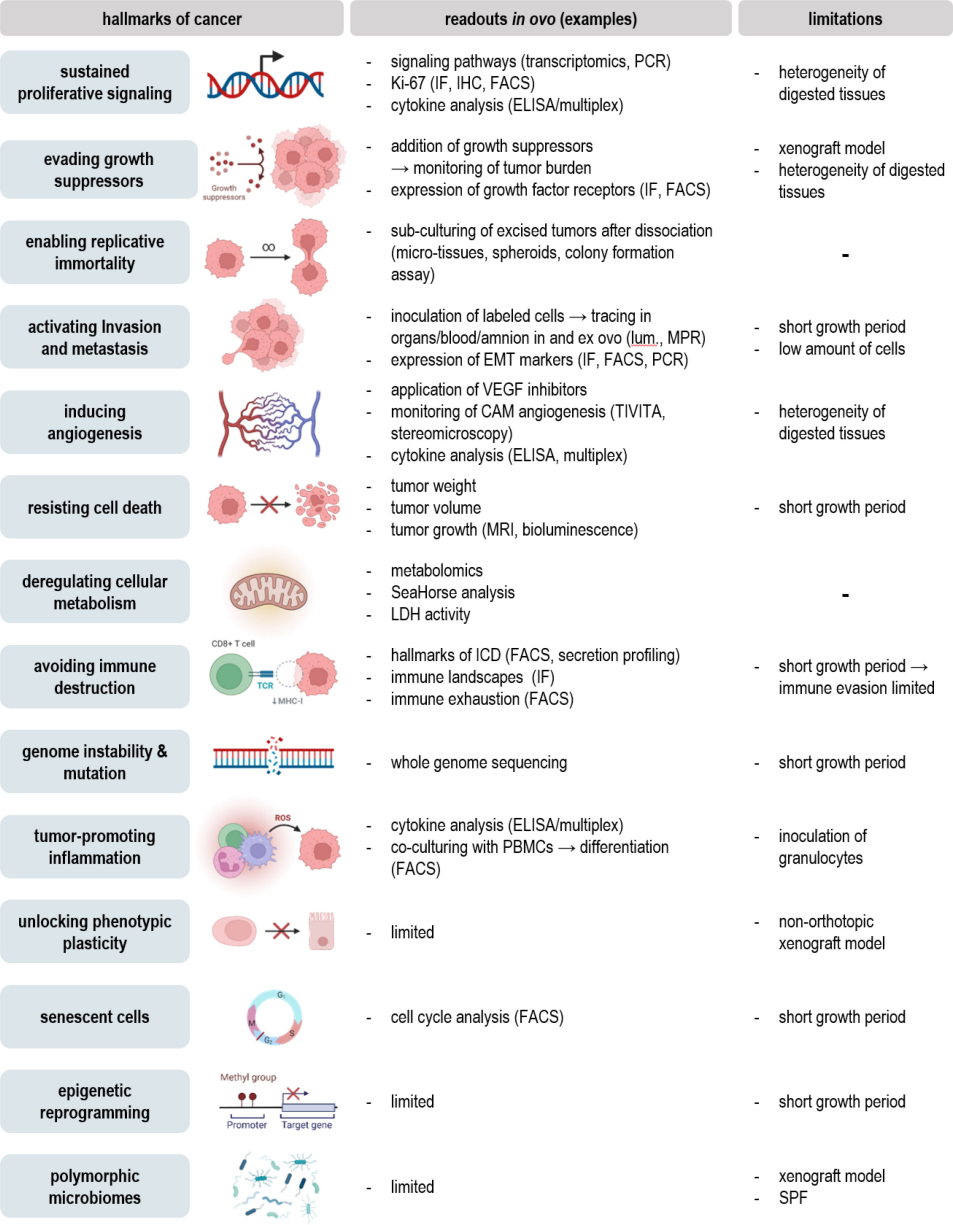
Addressing the hallmarks of cancer *in ovo*.

Evaluation of tumor growth, metastasis, and vascular remodeling *in ovo* can give important implications for the therapeutic efficacy and safety of novel agents or the influence of future targets based on knock-in/knock-out models. Notwithstanding, once the tumor is excised, an unlimited field of feasible downstream assays opens up (**Figure 3**). By that, detailed characterization of classical hallmarks of cancer can be achieved to provide mechanistic insights underlying oncogenesis or therapeutic effects (**Figure 4**).

Excised tumors can be embedded for further histological, immunohistochemical, or –fluorescent staining. By that, the architecture of the tumor microenvironment can be evaluated using conventional HE stainings (27), endothelial surface markers for evaluation of tumor perfusion (*view chapter 2.3*), or selective staining of chick and xenograft cells to evaluate their spatial distribution within the tumor (9). Staining of cleaved caspase 3 or TdT-mediated dUTP-biotin nick end labeling (TUNEL) (29) has been used to evaluate cell death in tissues combined with Ki-67 as a biomarker reflecting the proliferative state (15, 47). Various surface markers of interest, including integrins related to EMT (*view chapter 2.2*) (26), the immunogenicity of cell death, myeloid (28), and classical checkpoints (75) (*view chapter 3.3*) as well as diagnostic (9, 15, 35, 45) and predictive biomarkers (14, 31, 73) can be assessed. Tumor dissociation using adequate dissociation kits can be used to validate previous results at single cell levels using flow cytometry after intra- and extracellular staining with high throughput, including intracellular ROS levels, translocation, and phosphorylation of transcription factors, and an unlimited range of surface markers (13). Characterization of the TME can moreover be achieved using ELISA or bead-based multiplex assays for quantification of cytokine, chemokine, and growth factor release (26). As a consequence of aggressive growth, tumor cells exhibit a deregulated metabolism with shifts in carbon consumption characterized by high glycolytic activity, referred to as the Warburg effect (76). In the context of metabolic reprogramming during embryonal development, adaptions characteristic for aerobic glycolysis in cancer have been investigated by uptake measurements of fluorescent glucose analogs, such as 2-NBDG, basal oxygen consumption rates, and extracellular acidification rates using Seahorse technology, or conventional lactate and glucose uptake assay kits *in ovo* recently (77). Several studies have further focused on metabolic profiling *via* mass spectrometry (33, 34) or positron emission tomography (PET) imaging for evaluation of glucose metabolism and protein synthesis (53). At the simplest, the metabolic activity can be assessed by conventional colorimetric or fluorescence-based methods, including resazurin, MTT, and WST assays before or after tumor dissociation (10). While non-malignant cells exhibit a limited number of divisions, cancer cells bypass this barrier by hijacking telomerases to extend the length of their telomeres. Replicative immortality can be evaluated *in ovo* by sub-culturing of excised tumors after dissociation in the context of microtissues, spheroids, or conventional colony formation assays (78). Molecular insights into the mechanistic action of novel therapeutic agents, oncogenesis, and tumor progression have been achieved using western blot, PCR, and transcriptomics (79). Antibody panels with high specificity for chicken tissues based on the complete characterization of the chick genome enable to investigate interactions between xenograft and chicken tissues and help to distinguish between both.

## Aspects in cancer immunology

Experimental studies in animal models, rodents in particular, have yielded fundamental insights into key aspects of the development and regulation of the human hematopoietic and immune systems. However, as 65 million years of evolution may suggest, many aspects of mammalian biological systems, particularly their immune systems, display distinct differences (80). As therapeutic approaches become ever more sophisticated and specifically targeted, it becomes increasingly important to address the limitations of extrapolating pre-clinical discoveries to humans using animal models that more closely recapitulate human biological systems. Since the early 2000s, key advances have been made in the development of immunodeficient mice for generating humanized mice based on the mutant *IL2rγ* gene introduced in non-obese diabetic (NOD)/severe combined immunodeficiency (SCID) (81) and RAG1/2^null^ mice (82). Xenografting of human primary hematopoietic cells and tissues generating a functional human immune system became feasible, opening up novel avenues in basic and translational preclinical research, permitting insights into cause and cure of human diseases, including cancer immunity. As a major drawback, rodent models, and humanized mice in particular, are accompanied by ethical, cost, and efficiency constraints, limiting widespread adoption and pre-clinical investigation at larger scales. As a naturally immunodeficient host, the chick embryo model has served as an ideal preclinical alternative to rodent tumor models for almost a century. Engrafting of human immortalized cell lines and patient-derived xenografts already featuring a complex and unique microenvironment has successfully been applied. However, few studies have taken advantage of a naturally immunodeficient host to study tumor-immune responses without species-specific restrictions *in ovo.*

### Development of the avian immune system

Chicken embryos are naturally immunodeficient, enabling functional analysis of exogenously applied molecules, engrafted cells, or tissues without species-specific restrictions. Knowledge on the avian immune system and its responses still lag behind better studied mammalian biomedical systems, but the chicken genome sequence provided far better understanding in the past years. Development of the innate immune system takes place from ED3 until ED16. Avian macrophages are present in the circulatory system from ED4 and liver and spleen after ED12, where they become functional around ED14 (83). Studies on chicken macrophage receptor repertoires indicated the expression of scavenger, complement, Fc, C-type lectin, and mannose receptors, all essential for antigen recognition. During ontogenesis, embryonic macrophages play an important role in guiding the avian lymphoid system (84) and phagocytose cellular debris (85). As in humans, IFNγ can induce polarization into the pro-inflammatory M1 phenotype, while IL4 induces M2 polarization, involved in tissue repair and angiogenesis *via* secretion of IL10, TGFβ and VEGF (86, 87). Many aspects of mature dendritic cells (DCs), including their subsets, morphology, surface receptors and dedicated functions, share remarkable similarities between chickens and humans (88, 89). Their precursors have been detected in the avian thymus by ED11, but the time frame of maturation is still subject of investigations (88). Strikingly, while other secondary lymphoid organs vary only slightly in structure and morphology, birds lack encapsulated lymph nodes and the location of avian antigen presentation to lymphocytes remains unclear until now. Analog to mammalian neutrophils, chick embryos develop heterophils (90) containing lysosomal and non-lysosomal enzymes important for pathogen defense. Yet it is unclear whether heterophils represent a homogenous population or a subgroup of cells with different functionalities (91). Functional Natural Killer (NK) cells have been detected in the embryonic spleen starting from ED14. NK cell frequency in peripheral blood lymphocytes is lower in chicken (0,5-1%) compared to humans (2-5%) (92, 93) but identification of several receptors homologue to human KIR, NKp46 and LILR (91, 94) suggested the chicken NK cell biology generally to be close to mammals (95).

Primary lymphoid organs include the thymus and bursa of Fabricius, which are colonized by hematopoietic stem cells to become immunologically competent T and B cells before re-entering the circulation and colonizing peripheral lymphoid organs, broadly similar to mammalian immune systems. Early lymphoid cells deriving from the yolk sac and spleen are present in the thymus starting from ED8 and the bursa of Fabricius on ED11. Like mammals, avian T cells recognize MHC presented antigens *via* the heterodimeric T cell receptor (TCR), subdivided in αβ and γδ TCRs. Precursor hematopoietic cells enter the thymus in three waves, until TCR-γδ ^+^ and TCR-αβ_1_^+^ mature T cells migrate to the spleen by ED15 and ED19, respectively (96). Mature B cells leave the bursa of Fabricius only post-hatch (97). Generation of the avian antibody repertoire relies on somatic gene conversion, a process taking place during bursal development, as chickens only have a single copy of functional variable (V) and joining (J) segments for both chains of immunoglobulins (95, 98, 99), representing a major difference compared to mammals.

Although the avian immune system can respond to tumor cells by infiltration of monocytes and inflammatory-like cells such as avian heterophils, it is incapable of mounting an immune response before ED18. A non-specific inflammatory reaction has been reported if the experiment extends beyond ED15 but is dampened if the xenografts are implanted early during development when the avian immune system is still immature (100). Notwithstanding, due to ethical restrictions, *in ovo* experiments are widely terminated before cell-mediated immunity occurs.

### Addressing cancer immunity *in ovo*

The history of immune-oncological research *in ovo* is surprisingly short. Until now, two comprehensive studies present in the literature addressed tumor-immune cell interactions related to immune contexture in pancreatic ductal carcinoma (43) and reactive oxygen species-based therapy approaches *in ovo* (101) (**Table 3**). Other studies focused on the role of immune cells in inflammation-induced angiogenesis, partially translating results to the angiogenic switch in cancer (*view chapter 2.3*).

**Table 3:** Studies that focused on tumor-immune cell interactions *in ovo*.

Tumor cell line	Immune cell type	Immune cell origin	Species	E:T Ratio	Read-out	Reference (DOI)
PDA6606 (pancreas)	RAW264.7 (macrophage cell line)	immortalized	murine	1:1	- macroscopic remodeling (angiogenesis, matrix) - MRI - weight - flow cytometry - immunofluorescence	(43)
HT-29 (colon)	moDCs (from donor monocytes)	PBMCs	human	1:1	- weight	(101)
Panc-01 (pancreas)						
SKOV-3 (ovarian)						

In this view, Naldini and colleagues provided evidence that osteopontin (OPN) up-regulation in endothelial cells could represent a mechanism of amplifying growth factor-induced neovascularization *via* mononuclear phagocyte recruitment and increased levels of monocyte-derived pro-angiogenic cytokines. Administration of supernatants of human OPN-treated monocytes was highly angiogenic when delivered on the CAM but completely abrogated by neutralizing human anti-IL1 antibodies (102). Likewise, Huang and colleagues validated the anti-inflammatory and pro-angiogenic properties of human mesenchymal stem cell-derived exosomes^miRNA-21-5p^ for their application to promote ischemic tissue repair (103) and Pacini and colleagues investigated the role of human Gc protein-derived macrophage activating factor (GcMAF) in the context of deregulated angiogenesis in cancer *in ovo* (104). Ardi and colleagues addressed the contribution of human neutrophils and separately administered human neutrophil-derived MMP9 during the angiogenic switch in cancer *in ovo* (105), while Bansal and colleagues engrafted bone marrow-derived cells, peripheral blood mononuclear cells, and splenocytes isolated from C57BL/6 mice in the presence of various angiogenic growth factors in the context of choroidal neovascularization in acute macular degeneration (106). Although only partially related to oncogenesis, those findings provided evidence that hematopoietic precursors and mature immune cells of both myeloid and lymphoid lineage could successfully be implanted on the CAM, shaping a complex microenvironment through matrix and vascular remodeling, emphasizing the applicability to study tumor-immune cell interactions *in ovo*.

In the course of studying plasticity of tumor-associated macrophages, Khabipov and colleagues were the first to engraft immortalized RAW264.7 murine macrophages in a coculture model of pancreatic cancer *in ovo*. In a hydrogel onplant, murine PDA6606 pancreatic cancer cells were inoculated either with non-stimulated (naïve, M0) or pre-stimulated (polarized; M2) RAW264.7 macrophages at a 1:1 effector-target ratio, with 1 mio. cells, respectively. Pre-stimulated macrophages were generated by exposing naïve RAW264.7 cells to pre-conditioned PDA6606 supernatants 1:2 in fresh medium for 72 h. Interestingly, MRI measurements revealed pre-stimulated macrophages to increase PDA6606 tumor growth compared to monoculture tumors, while naïve did not. The authors hypothesized that the short time span (72 h) limited M2 polarization in naïve macrophages during tumor development (*view chapter 3.3*). Flow-cytometric analysis of dissociated PDA6606:RAW264.7(pre-stimulated) co-culture tumors confirmed the M2-phenotype of the latter, while tissue sections emphasized their role in promoting angiogenesis and matrix remodeling in pancreatic cancer (43). The second study focused on redox-based effects of oxidant-enriched carrier solutions for adjuvant peritoneal lavage in the context of peritoneal carcinomatosis. *In vitro*, the approach increased immunogenicity and uptake of three human carcinoma cell lines by monocyte-derived dendritic cells. The authors used the *in ovo* model as a screening platform to validate the therapeutic efficacy observed *in vitro* in a physiologically more complex model. Here, human monocyte-derived dendritic cells isolated from healthy donors were engrafted 1:1 with the immortalized tumor cell lines and grown on the CAM for 7 days. Reduction in tumor burden correlated well with results obtained in a model of peritoneal carcinomatosis in mice, emphasizing the translational relevance of the chicken embryo model for pre-clinical studies (101). A widely different approach was employed by Wang and colleagues, who took advantage of homologies in the PD1/PDL1 axis between chickens and humans and proposed the TUM-CAM model as an alternative for immunooncological drug development. Clinically approved checkpoint inhibitors mitigated tumor growth *in ovo* and partially restored T cell-mediated tumor toxicity of chicken lymphocytes *in vitro* (75). However, appropriate controls and quantification of crucial mechanistic insights, including checkpoint antibody binding assays, were largely missing, questioning the scientific relevance of such findings. Furthermore, tumors were excised at ED18, only shortly after mature T cells could be detected in the avian circulatory system (*view chapter 3.2*).

### Obstacles or chance?

The caveat of immunooncological research in the chicken embryo model relies in the definition of an appropriate study design depending on the scientific question and careful data interpretation with respect to model-specific characteristics. Comprehensive studies are needed to establish and evaluate eligible protocols to investigate cancer immunity *in ovo*. Key steps, including reasonable timing of immune cell grafting and convenient routes, co-administration of cytokines and growth factors, and lineage-dependent differences have to be clarified. In some cases, additional irradiation to destroy the developing avian immune system might be worth considering.

Importantly, the majority of *in ovo* experiments end, due to ethical restrictions in most countries, with ED14, before mature T and B cells, macrophages, and NK cells have been detected in the avian circulation. The onset of DC maturation is yet unknown (83, 95). Future studies have to clarify the role of remaining host immune cell precursors in the outcome and evaluation of tested immunotherapies *in ovo*. Likewise, it is conceivable that evolutionarily conserved mechanisms or higher homology protein structures are more involved in the control of tumor growth in this system due to higher ligand/receptor cross-reactivity compared to species-specific mechanisms. Many of the hallmarks of cancer recapitulate unicellular modalities, and their onset is suggested to correlate inversely with the chronological sequence in which the respective genes evolved (107, 108). Comparative gene expression profiling helped to identify the repertoire of avian immune receptors, surface-expressed antigens, cytokines, and growth factors and link them to mammalian orthologs in the past. Cross-reactivity between cytokines from different species has been shown to appear with an apparent threshold of around 60% amino acid identity, in dependence on the folding family. However, despite similar biological activities, chicken cytokines have only 25 – 35% amino acid identity with their mammalian counterparts (109, 110). The role of cross-reactivity in the xenogeneic system remains unclear as of now and has to be elucidated in future studies. In analog to the different models of humanization in mice, this review suggests the dual engraftment of human immune and tumor cells in fertilized chicken embryos as a xenogeneic system to study immunotherapies in a more comprehensive but cost-efficient semi *in vivo* model. This would include the engraftment of human leucocytes, similar to the human peripheral blood leucocyte (Hu-PBL) model in mice. In the Hu-PBL model, a major caveat remains the variable efficacy of different immune cell subpopulations to be engrafted in humanized mice. As such, only low levels of human B lymphocytes and myeloid cells have generally been detected in the circulatory system, most likely due to the lack of human cytokines required for survival (111). Along similar lines, the longevity and functionality of different human immune cell subpopulations and the need for coadministration of relevant human cytokines to ensure their survival has to be evaluated *in ovo.*

**Figure 5:**
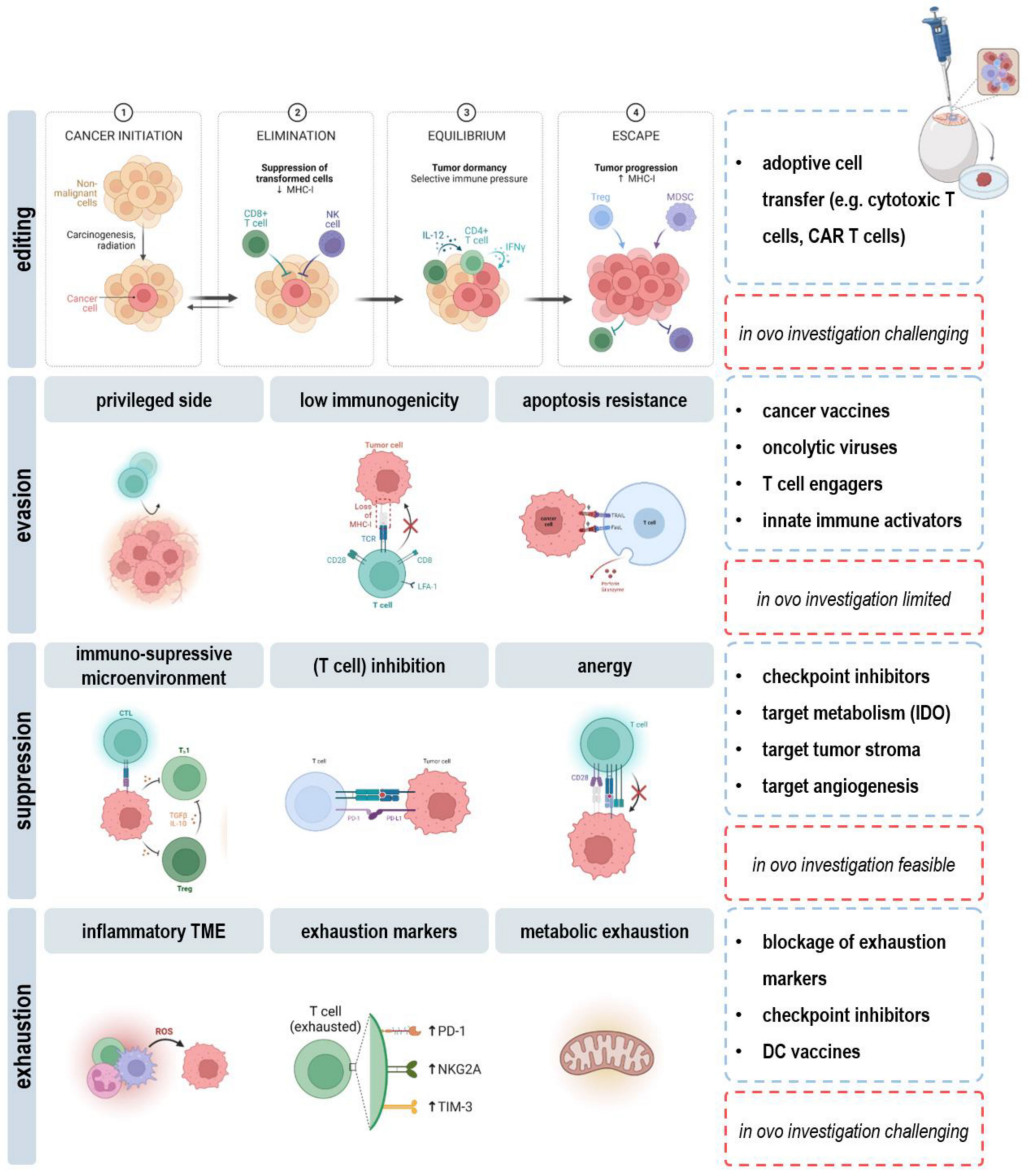
Applicability and limitations of the *in ovo* model in immuno-oncological research.

As a major drawback, the short experimental time span dictates limitations in long-term evaluation of cancer immunity *in ovo*. As such, the dual role of the immune system in the context of immunoediting cannot be addressed. Rare tumor cell variants persisting after the elimination and equilibrium phase become clinically apparent if altering their response to immunoselection pressures in the escape phase (112). Here, the humanized chicken embryo model can provide insights in underlying mechanisms or the efficacy of novel therapeutic strategies using the broad range of applicable read-outs discussed in this review (*view chapters 2.2-2.4*). For instance, downregulation or loss of tumor antigens, antigen-presenting machinery, or lack of costimulatory molecules results in ineffective priming and activation of DCs and CD8^+^ T cells in the tumor microenvironment. Therapeutic approaches aim to turn the tide by induction of immunogenic cell death (113), engineering T cells to recognize specific antigens on the surface of cancer cells (114), or administration of adjuvants such as CD40 agonists and anti-CD137 antibodies (115) and can readily be explored *in ovo*. Another obstacle with tumor cells is their ability to upregulate resistance mechanisms against cytotoxic effectors of immunity, e.g., *via* STAT3 signaling or increased expression of pro-survival and growth factor genes, including Bcl-2, Her2, EGFR, and c-kit (116, 117). Targeted therapies that find and kill cancer cells by homing in on molecular changes in respective oncogene and growth receptor pathways can likewise be administered and evaluated *in ovo*. Moreover, specific molecular changes can be identified to develop novel agents targeting transformed cells in the future. Despite fooling the immune system in dangerous hide and seek, cancer cells hijack their surrounding environment to create an immune barrier, cause immune cell malfunction, or even co-opt and modify innocent bystanders to produce tumor promoting growth factors, chemokines and matrix-degrading enzymes. In this view, therapies targeting cytokines (e.g., VEGF, TGFβ), metabolic factors (e.g., adenosine, PGE_E2_), or blockage of inhibitory receptors (e.g., CTLA4, PD1, TIM3) on effector cells can be considered. Investigation of functional orientation, density, and spatial distribution further allows for correlation of complex immune contexture with tumor growth and outcome (**Figure 5**).

Overall, extensive research is needed to clarify if the *in ovo* model represents a faithful avatar for evaluating immunotherapies in preclinical oncological research. Nonetheless, considering previous research in humanized *in ovo* models of inflammation-induced angiogenesis and latest reports studying tumor-immune cell interactions *in ovo*, it is conceivable that fertilized chicken embryos could represent a comprehensive research model in immunooncological research in the future.

## Outlook

A major advantage relies in the CAM’s easy accessibility and low rejection rate to facilitate increasing the model’s complexity by engrafting 3D spheroids (118, 119) or multicellular organoids. For instance, mature organoids derived from human-induced pluripotent stem cells (PSCs) rapidly connected to the vascular network of the chick embryo after transferring them on the CAM (120), and PSC–derived inner ear and kidney organoids demonstrated the model’s potential to optimize developmental maturity and functionality of organoids based on vascularization (121, 122). Along similar lines, grafting of tumor organoids, also combined with immune cells and/or fibroblasts, reflecting tissue heterogeneity in cancer to a greater extent, can be employed (123).

**Figure 6:**
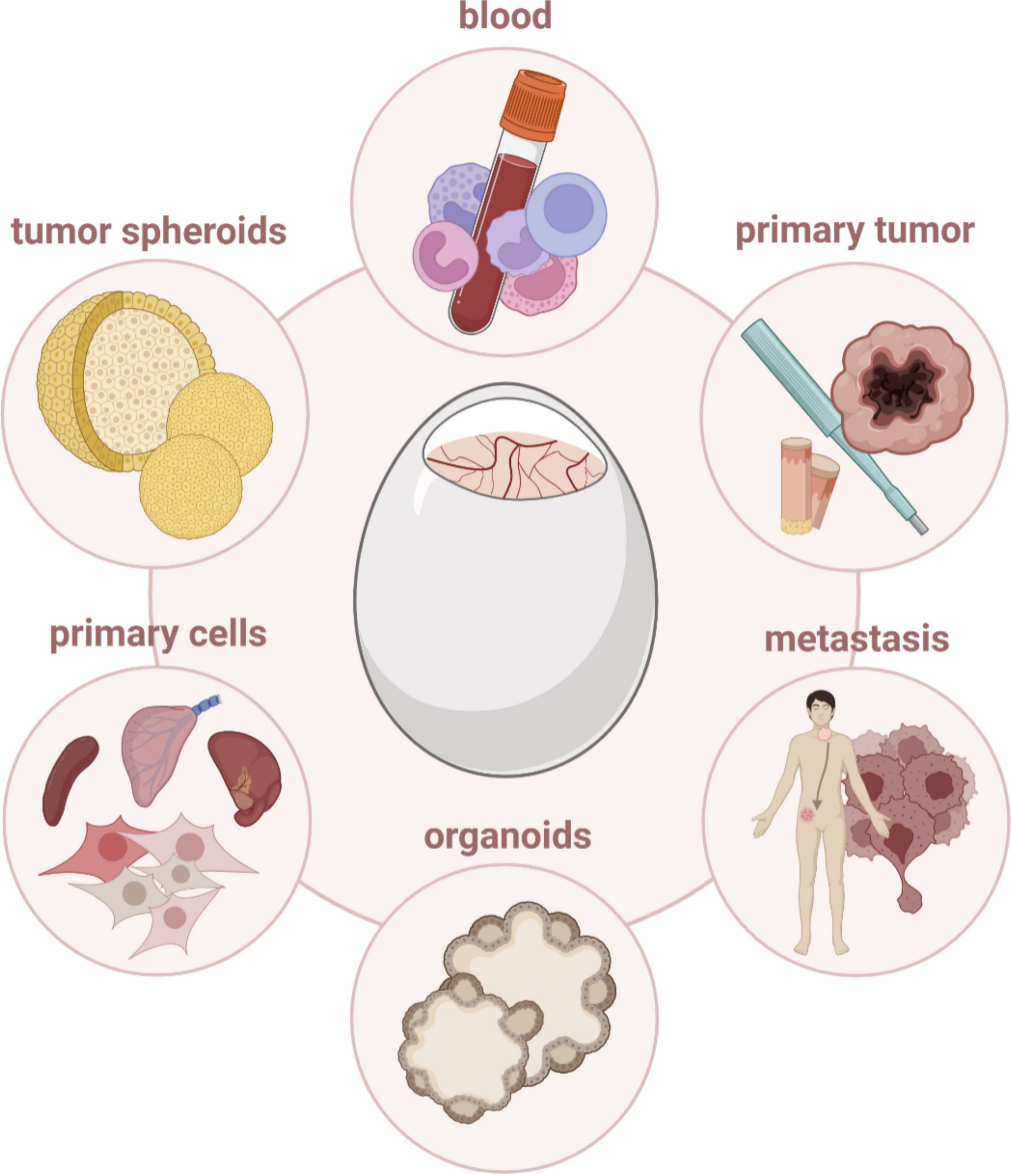
Cells and tissues that can be engrafted *in ovo*.

Engrafting patient-derived xenografts (PDX) could provide improved diagnostic opportunities, tailoring medical treatments in the context of personalized medicine. PDXs preserve many features of primary tumor biology and heterogeneity, including genetic, proteomic, morphological, and pharmacologic characteristics. Moreover, PDXs are characterized by their unique immunological history with cancer-specific immune evasion mechanisms and substantial heterogeneity in (immune) microenvironments (124), reflecting the clinical situation more accurately. Transplantation of human glioblastoma biopsies on the avian CAM emphasized their ability to recapitulate features of the primary tumor, including cellular polymorphism and infiltrating immune cells (125). Likewise, bladder, prostate, and nasopharyngeal carcinoma-derived xenografts could mimic tumor biology, growth, angiogenesis, extracellular matrix interaction, and metastasis as found in the patient (126, 127). Immunohistochemistry, analysis of mutational load, mRNA, and microRNA expression profiling unveiled primary PDAC tumors grown on the CAM to share histopathological and genetic characteristics with the parent tumors (18). Despite solid tumors, tumor cell engraftment and distribution of intravenously injected CD34^+^ leukemic cells were validated by transcript detection *via* reverse transcription polymerase chain reaction (RT-PCR) in blood, bone marrow, spleen, and liver from embryos (128). Circulating cancer stem cells were engrafted to analyze aggressiveness and proliferation capacity of primary tumors, closely resembling its complex structure for pre-clinical drug screenings and biomarker discovery (129). Re-grafting and repetitive passaging of human colorectal liver metastases on the CAM, paralleled by immunophenotyping and evaluation of invasiveness, enabled long-term monitoring of PDX tumors, displaying an interesting approach to study tumor progression *in ovo* (130) (**Figure 6**).

Needless to say, the chicken embryo model can clearly not be suggested as a complete replacement for conventional pre-clinical animal models. Despite other advantages, humanized mice models aim, e.g., for genetic modifications of HLA expression to ensure appropriate antigen presentation in peripheral tissues or transgenic expression of human cytokines to improve development and function of transplanted cells at large time scales, which widely outperforms the limits of studying oncogenesis and cancer immunity *in ovo.* On the contrary, the well-vascularized CAM provides high efficiency of tissue grafting, bridging the gap between *in vitro* and complex but costly mammalian *in vivo* models while supporting the 3Rs guidelines. With some major limitations and caveats to keep in mind, the humanized chicken chorioallantoic tumor model could serve as an alternative for pre-clinical immunooncological drug screenings and basic immunological research. It provides a quick, reproducible and effective evaluation of different therapeutic options and has, based on PDX, the potential for development of tailored treatments, including personalized immunotherapy (131).

## Author contributions

SB designed the review; LM and JB designed the figures; LM and JB performed experiments; all authors wrote the manuscript draft and reviewed the draft. All authors contributed to the article and approved the submitted version.

## Funding

Funding was received by the German Federal Ministry of Education and Research (BMBF, grant numbers to SB: 03Z22DN11 and 03Z22Di1).

## Acknowledgments

Figure design was supported by the biorender.com platform.

## Conflict of interest

The authors declare that the research was conducted in the absence of any commercial or financial relationships that could be construed as a potential conflict of interest.

## Publisher’s note

All claims expressed in this article are solely those of the authors and do not necessarily represent those of their affiliated organizations, or those of the publisher, the editors and the reviewers. Any product that may be evaluated in this article, or claim that may be made by its manufacturer, is not guaranteed or endorsed by the publisher.
